# Approaches for monitoring and treating cardiomyopathy among cancer survivors following anthracycline or thoracic radiation treatment

**DOI:** 10.1186/s40959-022-00138-x

**Published:** 2022-05-12

**Authors:** Arash Delavar, Catherine Boutros, Dana Barnea, Wendy L. Schaffer MD, Emily S. Tonorezos

**Affiliations:** 1 Department of Medicine, Memorial Sloan Kettering Cancer Center, New York, NY USA; 2 University of California San Diego School of Medicine, La Jolla, CA San Diego, USA; 3 City University of New York School of Medicine, New York, NY USA; 4 Tel Aviv Sourasky Medical Center, Tel Aviv, Israel; 5 Division of Cancer Control and Population Sciences, National Cancer Institute, National Institutes of Health, Rockville, MD USA; 6 Office of Cancer Survivorship, National Cancer Institute, 9409 Medical Center Drive, Rockville, MD 20850 USA

**Keywords:** Preventive cardiology, Cancer survivorship, Anthracycline, Radiation therapy, Cardiomyopathy, Medical decision making

## Abstract

**Background:**

Anthracycline chemotherapy and thoracic radiation therapy (RT) are known causes of cardiomyopathy among cancer survivors, however, management guidelines for this population are lacking. In this study we describe our single institution management approach for cancer survivors with low left ventricular ejection fraction (LVEF) secondary to cancer treatment.

**Methods:**

We conducted a retrospective descriptive study of childhood and young adult (CAYA) cancer survivors in the Adult Long-Term Follow-Up Clinic at Memorial Sloan Kettering Cancer Center enrolled between November 2005 and July 2019. Those included were treated with anthracycline and/or thoracic RT as a part of their cancer therapy and had recorded a LVEF of < 55% on at least one post-treatment echocardiogram. Details regarding survivor characteristics, screening, and management were abstracted. Differences in management approaches among survivors with LVEF of 50–54.9%, 40–49.9%, and < 40% were described. Qualitative management approaches were abstracted as well.

**Results:**

Among 668 CAYA survivors in the initial cohort, 80 were identified who had received anthracycline and/or thoracic RT and had a LVEF of < 55%. Median age at cancer diagnosis was 16.1 years, median time from cancer diagnosis was 25.8 years, and 55% of survivors were female. Cardiology referrals, nuclear stress tests, multi-gated acquisition scans, angiograms, echocardiograms, treatment with angiotensin converting enzyme inhibitors or receptor blockers, beta-blockers, diuretics, aldosterone antagonists, aspirin, and insertion of pacemaker or implantable cardioverter-defibrillators differed by LVEF category. Documentation suggested uncertainty regarding management of survivors with borderline low-LVEF, with low-LVEF that improved on follow-up, and with subsequent cancers requiring additional treatment.

**Conclusions:**

The management of CAYA cancer survivors with low-LVEF largely followed guidelines designed for the general population, however, uncertainty remains for issues specific to cancer survivors. Cardiomyopathy management guidelines that address issues specific to cancer survivors are needed.

## Introduction

Five-year survival rates for childhood, adolescent, and young adult (CAYA) cancer have increased from 58% in the mid-1970s to well over 80% in the past decade [1], helping to avert an estimated 38 032 cancer deaths from 1975 to 2006 [2]. It is estimated that there are nearly 400 000 survivors of childhood and adolescent cancer in the United States [3]. As more of these children live on to adulthood, cancer survivor health is an increasingly relevant aspect of healthcare. Cancer survivors have a higher risk of many chronic health conditions, including joint problems, infertility, hearing and vision loss, cognitive dysfunction, secondary cancers [4, 5], and cardiac problems – especially as a result of anthracycline chemotherapy or thoracic radiation therapy (RT) [5–7].

About 60% of CAYA survivors have been exposed to anthracyclines and/or thoracic RT as a part of their treatment [5]. Cardiac complications associated with these therapies are largely dependent on cumulative dose and time since treatment, with a relative hazard of up to six times that of siblings [8]. Several organizations, including the Children’s Oncology Group [9] and European Society of Cardiology (ESC) [10], provide guidelines for detecting cardiomyopathy in this population, with recommendations that include serial echocardiography [11]. However, once heart problems are detected, management guidelines for low left ventricular ejection fraction (LVEF) or cardiomyopathy specific to cancer survivors are lacking [12–14]. The ESC does recommend angiotensin converting enzyme inhibitors or receptor blockers (ACEI/ARB) in combination with beta-blockers in cancer survivors with symptomatic or asymptomatic cardiac dysfunction (unless contraindicated) but do not provide recommendations on other treatments [10]. A study using Delphi methodology querying physicians for their management approach of these survivors elucidated on this uncertainty, finding agreement in some areas (echocardiogram and ECG screening frequency, exercise promotion, referrals to cardiology, and use of ACEI) and disagreement in other areas (use of other cardiac testing, frequency of screening during pregnancy, and use of beta-blockers) [15].

In this retrospective descriptive study, we obtain data from the Memorial Sloan Kettering (MSK) Adult Long-Term Follow-Up Program to describe approaches for monitoring and managing low-LVEF in CAYA cancer survivors with a history of anthracycline chemotherapy and/or thoracic RT. For clarity, we categorized survivors by lowest LVEF on transthoracic echocardiogram, with a goal of elucidating how these survivors are managed and how everyday practice compares to guideline-concordant care for the general population [12, 13].

## Methods

### Study Population

Our study population consists of CAYA cancer survivors enrolled in the MSK Adult Long-Term Follow-Up Program enrolled between November 2005 and July 2019. The MSK Adult Long-Term Follow-Up Program delivers longitudinal risk-based health care, including management of late effects, for adults who had their first primary malignancy prior to age 40 and who are at risk for late effects or have multiorgan complications following cancer therapy [16]. Survivors must be over 18 and finished with therapy to be followed in the clinic. Screening and surveillance for late effects occurs during an annual visit. In accordance with the COG guidelines [9], an annual cardiac exam is performed including medical history and blood pressure recordings, as well as echocardiograms at intervals based on anthracycline and thoracic RT dose. From the initial cohort, survivors who received anthracycline and/or thoracic RT as part of their cancer treatment and had a lowest LVEF of < 55% on echocardiogram were identified.

### Main outcomes and measures

Patient characteristics, such as demographics, cancer diagnoses, comorbidities, and treatment details including anthracycline doses (converted to doxorubicin equivalents) [17] and thoracic RT doses were recorded. Detailed measures from the echocardiogram with the lowest recorded LVEF were obtained, including fractional shortening, left ventricular wall thickness, left atrial size, and valvular disease. Other heart health outcomes such as coronary artery disease, congestive heart failure, and myocardial infarction were obtained from patient charts. Additional cardiac monitoring was abstracted as well, including subsequent echocardiograms, ECGs, Holter monitors, stress tests, multi-gated acquisition (MUGA) scans, cardiac MRIs, and angiograms. Treatments provided, including medications such as ACEI/ARB, beta-blockers, diuretics, aldosterone antagonists, statins, and aspirin were obtained, as well as information on surgical procedures such as pacemaker or implantable cardioverter-defibrillator (ICD) placement, stent placement, valve replacement, and coronary artery bypass. Lastly, qualitative information on assessments and plans were also obtained to elucidate on the medical management of this unique patient population.

Results were stratified into three lowest recorded LVEF categories: < 41%, 41–49.9%, and 50–54.9%, which were informed by American Heart Association guidelines [13] representing survivors that have clearly reduced LVEF, are considered borderline, or may need additional cardiac monitoring, respectively.

## Results

### Overview

Among 668 survivors who received anthracycline chemotherapy and/or thoracic RT, 80 (12.0%) had a lowest recorded LVEF of < 55%. Of these 80 survivors, 70 (87.5%) were non-Hispanic White and 36 (45.0%) were male. The most common primary cancer types represented were 31 (38.8%) with Hodgkin’s lymphoma, 26 (32.5%) with sarcoma, and 12 (15.0%) with non-Hodgkin’s lymphoma. Seventy-three (91.3%) survivors received anthracycline chemotherapy and 49 (61.3%) received thoracic RT as part of their cancer treatment. Of those who received anthracycline chemotherapy, 10 (12.5%) received dexrazoxane as a cardioprotective measure. Fifty-seven (71.3%) were diagnosed before age 20, and 69 (86.3%) were living by the end of the follow-up period (Table 1). Survival status (*P* = 0.027), diabetes mellitus (*P* = 0.004), thoracic RT dose (*P* = 0.030), and use of dexrazoxane (*P* = 0.049) were significantly varied by group.

**Table 1:** Characteristics of 80 childhood and young adult cancer survivors with low left ventricular ejection fraction

Characteristic	N (%)	LVEF 50–54.9% *N* = 38 (47.5%)	LVEF 41–49.9% *N* = 18 (25.0%)	LVEF < 41% *N* = 24 (30.0%)	*P*-value^a^
Survival status					0.027
Alive	69 (86.3)	36 (94.7)	16 (88.9)	17 (70.8)	
Deceased	11 (13.8)	2 (5.3)	2 (11.1)	7 (29.2)	
Age at diagnosis					0.706
0–5	12 (15.0)	4 (10.5)	3 (16.7)	5 (20.8)	
6–10	8 (10.0)	4 (10.5)	3 (16.7)	1 (4.2)	
11–15	18 (22.5)	9 (23.7)	3 (16.7)	6 (25.0)	
16–20	19 (23.8)	9 (23.7)	6 (33.3)	4 (16.7)	
> 20	23 (28.8)	12 (31.6)	3 (16.7)	8 (33.3)	
Sex					0.995
Male	36 (45.0)	17 (44.7)	8 (44.4)	11 (45.8)	
Female	44 (55.0)	21 (55.3)	10 (55.6)	13 (54.2)	
Race/ethnicity					0.223
NH White	70 (87.5)	32 (84.2)	17 (94.4)	21 (87.5)	
NH African American	5 (6.3)	4 (10.5)	0 (0.0)	1 (4.2)	
Hispanic (any race)	3 (3.8)	2 (5.3)	0 (0.0)	1 (4.2)	
NH Asian	1 (1.3)	0 (0.0)	0 (0.0)	1 (4.2)	
Other	1 (1.3)	0 (0.0)	1 (5.6)	0 (0.0)	
Comorbidities					
Diabetes	12 (15.0)	1 (2.6)	3 (16.7)	8 (33.3)	0.004
Hypertension	12 (15.0)	6 (15.8)	1 (5.6)	5 (20.8)	0.383
Hyperlipidemia	22 (27.5)	10 (26.3)	5 (27.8)	7 (29.2)	0.970
Primary Cancer					0.095
Hodgkin's lymphoma	31 (38.8)	9 (23.7)	7 (38.9)	15 (62.5)	
Non-Hodgkin's lymphoma	12 (15.0)	6 (15.8)	3 (16.7)	3 (12.5)	
Leukemia	5 (6.3)	3 (7.9)	1 (5.6)	1 (4.2)	
Sarcoma	26 (32.5)	18 (47.4)	5 (27.8)	3 (12.5)	
Neuroblastoma	3 (3.8)	1 (2.6)	2 (11.1)	0 (0.0)	
Retinoblastoma	1 (1.3)	1 (2.6)	0 (0.0)	0 (0.0)	
Germ cell cancer	1 (1.3)	0 (0.0)	0 (0.0)	1 (4.2)	
Renal cancer	1 (1.3)	0 (0.0)	0 (0.0)	1 (4.2)	
Treatment type					0.008
Anthracycline only	31 (38.8)	21 (55.2)	4 (22.2)	6 (25.0)	
RT only	7 (8.8)	0 (0.0)	2 (11.1)	5 (20.8)	
Anthracycline and RT	42 (52.5)	17 (44.7)	12 (66.7)	13 (54.2)	
Anthracycline dose (mg/m^2^)^b^					0.191
None	7 (8.8)	0 (0.0)	2 (11.1)	5 (20.8)	
< 250	16 (20.0)	7 (18.4)	5 (27.8)	4 (16.7)	
250–399	31 (38.8)	16 (42.1)	6 (33.3)	9 (37.5)	
400–500	17 (21.3)	10 (26.3)	4 (22.2)	3 (12.5)	
> 500	5 (6.3)	3 (7.9)	0 (0.0)	2 (8.3)	
Dose unknown	4 (5.0)	2 (2.6)	1 (5.6)	1 (4.2)	
Thoracic RT dose (cGy)					0.030
None	31 (38.8)	21 (55.3)	4 (22.2)	6 (25.0)	
< 2500	14 (17.5)	6 (15.8)	5 (27.8)	3 (12.5)	
2500–3999	18 (22.5)	8 (21.1)	3 (16.7)	7 (29.2)	
4000–6000	12 (15.0)	2 (5.3)	5 (27.8)	5 (20.8)	
> 6000	2 (2.5)	0 (0.0)	0 (0.0)	2 (8.3)	
Dose unknown	3 (3.8)	1 (2.6)	1 (5.6)	1 (4.2)	
Dexrazoxane therapy	10 (12.5)	9 (23.7)	1 (5.6)	0 (0.0)	0.049

*LVEF* Left ventricular ejection fraction, *N* Number, *NH* Non-Hispanic, *mg* milligrams. *m* meters, *cGy* centigray

^a^Calculated from chi-square tests assessing differences in outcomes between LVEF categories. Statistical tests were two-tailed

^b^Anthracycline doses were converted to doxorubicin equivalents

The mean lowest LVEF on echocardiogram was 44.7%. Twenty-four survivors (30%) had a lowest LVEF of < 41% and 6 (7.5%) had fractional shortening of < 20% on lowest LVEF echocardiogram. The most common valvular diseases included 24 (30.0%) with mitral regurgitation and 28 (35.0%) with tricuspid regurgitation. Twenty-three survivors (28.8%) had symptoms associated with low-LVEF, 21 (26.3%) were diagnosed with coronary artery disease, 14 (17.5%) were diagnosed with congestive heart failure, and 2 (2.5%) died of a cardiac complication by the end of the follow-up period (Table 2). Low fractional shortening (*P* < 0.001), left ventricular hypertrophy (*P* = 0.013), left atrial enlargement (*P* = 0.003), mitral regurgitation (*P* < 0.001), tricuspid regurgitation (*P* = 0.011), aortic regurgitation (*P* = 0.007), coronary artery disease (*P* = 0.033), and congestive heart failure (*P* < 0.001) were significantly varied by group.

**Table 2:** Cardiac health measures among 80 childhood and young adult cancer survivors with low left ventricular ejection fraction

Test	N (%)	LVEF 50–54.9% *N* = 38 (47.5%)	LVEF 41–49.9% *N* = 18 (25.0%)	LVEF < 41% *N* = 24 (30.0%)	*P*-value^a^
Fractional shortening (%)					< 0.001
> 25	37 (46.3)	31 (81.6)	3 (16.7)	3 (12.5)	
20–25	16 (20.0)	2 (5.3)	9 (50.0)	5 (20.8)	
< 20	6 (7.5)	0 (0.0)	0 (0.0)	6 (25.0)	
Missing	21 (26.3)	5 (13.2)	6 (33.3)	10 (41.7)	
Left ventricular hypertrophy	8 (10.0)	1 (2.6)	1 (5.6)	6 (25.0)	0.013
Left atrial enlargement	7 (8.8)	1 (2.6)	0 (0.0)	6 (25.0)	0.003
Valvular disease					
Mitral regurgitation	24 (30.0)	5 (13.2)	4 (22.2)	15 (62.5)	< 0.001
Tricuspid regurgitation	28 (35.0)	8 (21.1)	6 (33.3)	14 (58.3)	0.011
Aortic regurgitation	9 (11.3)	0 (0.0)	3 (16.7)	6 (25.0)	0.007
Pulmonic regurgitation	13 (16.3)	6 (15.8)	1 (5.6)	6 (25.0)	0.238
Mitral stenosis	2 (2.5)	1 (2.6)	0 (0.0)	1 (4.2)	0.692
Aortic stenosis	6 (7.5)	2 (5.3)	2 (11.1)	2 (8.3)	0.727
Symptomatic at lowest ejection fraction	23 (28.8)	8 (21.1)	5 (27.8)	10 (41.7)	0.216
Coronary artery disease	21 (26.3)	7 (18.4)	3 (16.7)	11 (45.8)	0.033
Congestive heart failure	14 (17.5)	1 (2.6)	3 (16.7)	10 (41.7)	< 0.001
Myocardial infarction	7 (8.8)	1 (2.6)	2 (11.1)	4 (16.7)	0.150
Cardiac-related death	2 (2.5)	1 (2.6)	1 (5.6)	0 (0.0)	0.600

*LVEF* Left ventricular ejection fraction, *N* number

^a^Calculated from chi-square tests assessing differences in outcomes between LVEF categories. Statistical tests were two-tailed

**Fig. 1:**
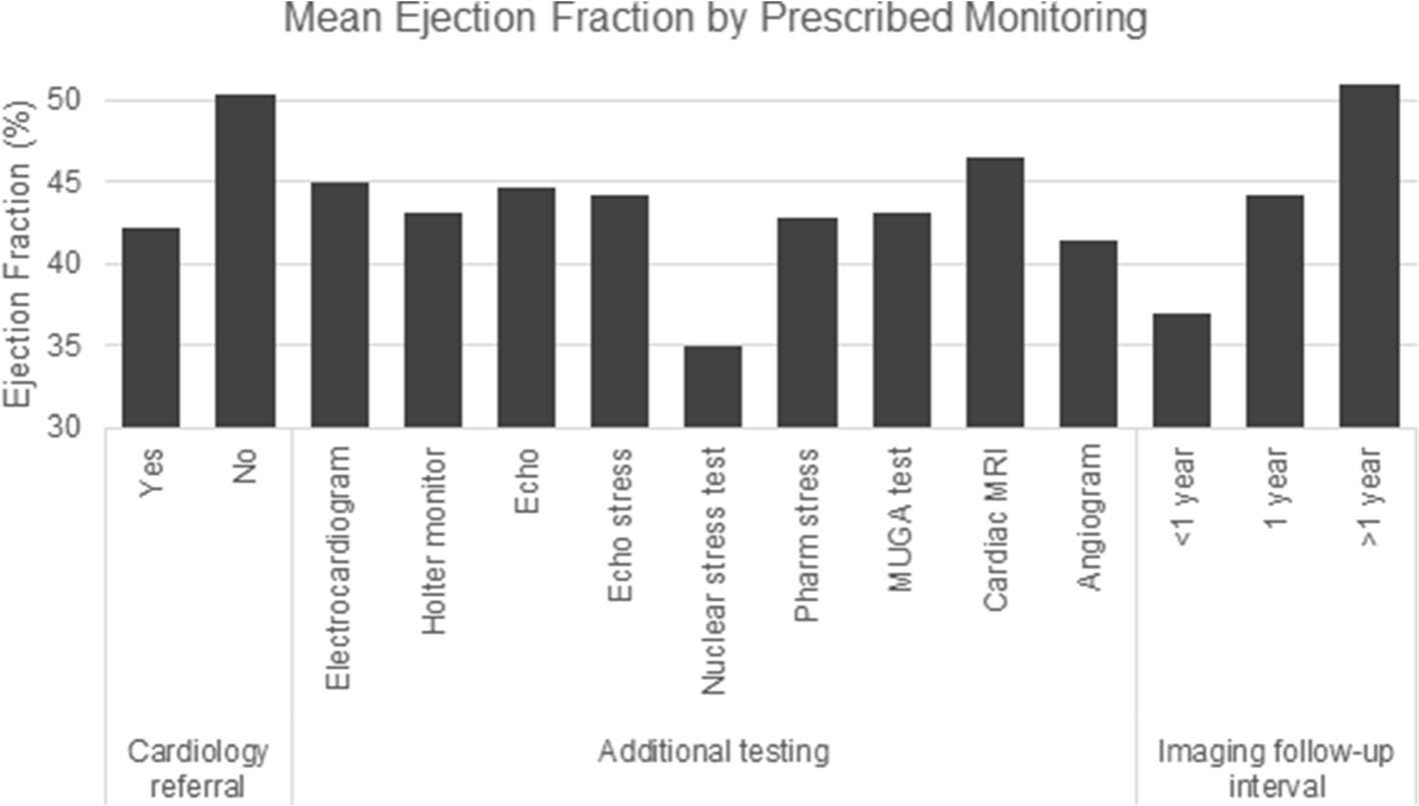
Mean ejection fraction by prescribed monitoring among 80 childhood and young adult cancer survivors with low left ventricular ejection fraction

Fifty-six survivors (70.0%) overall received a referral to a cardiologist, and 77 (96.3%) received additional cardiac testing after their lowest recorded LVEF. The most common subsequent testing included 71 (88.8%) with at least one ECG, 26 (32.5%) with at least one echocardiogram stress test, and 22 (27.5%) with at least one angiogram. Sixty-two (77.5%) survivors had echocardiogram intervals of 1 year, 12 (15.0%) with intervals of > 1 year, and 6 (7.5%) with intervals of < 1 year (Table 3). Cardiology referrals (*P* = 0.003), nuclear stress tests (*P* = 0.030), MUGA scans (*P* = 0.027), angiograms (*P* = 0.005), and shorter echocardiogram follow-up intervals (*P* < 0.001) were significantly varied by group. Mean LVEFs by prescribed cardiac monitoring ranged from 34.6% for nuclear stress tests to 46.6% for cardiac MRI (Fig. 1).

**Table 3:** Cardiac monitoring among 80 childhood and young adult cancer survivors with low left ventricular ejection fraction

Monitoring	N (%)	LVEF 50–54.9% *N* = 38 (47.5%)	LVEF 41–49.9% *N* = 18 (25.0%)	LVEF < 41% *N* = 24 (30.0%)	*P*-value^a^
Cardiology referral	56 (70.0)	20 (52.6)	14 (77.8)	22 (91.7)	0.003
Additional testing					
Any test	77 (96.3)	38 (100.0)	16 (88.9)	23 (95.8)	0.123
Electrocardiogram	71 (88.8)	35 (92.1)	14 (77.8)	22 (91.7)	0.246
Holter monitor	10 (12.5)	4 (10.5)	1 (5.6)	5 (20.8)	0.293
Echocardiogram stress test	26 (32.5)	10 (26.3)	8 (44.4)	8 (33.3)	0.398
Nuclear stress test	5 (6.3)	0 (0.0)	1 (5.6)	4 (16.7)	0.030
Pharmacological nuclear stress test	4 (5.0)	1 (2.6)	2 (11.1)	1 (4.2)	0.387
MUGA scan	4 (5.0)	0 (0.0)	3 (16.7)	1 (4.2)	0.027
Cardiac MRI	18 (22.5)	9 (23.7)	4 (22.2)	5 (20.8)	0.966
Angiogram	22 (27.5)	4 (10.5)	7 (38.9)	11 (45.8)	0.005
Echocardiogram follow-up interval					< 0.001
< 1 year	6 (7.5)	0 (0.0)	1 (5.6)	5 (20.8)	
1 year	62 (77.5)	27 (71.1)	17 (94.4)	18 (75.0)	
> 1 year	12 (15.0)	11 (28.9)	0 (0.0)	1 (4.2)	

*LVEF* Left ventricular ejection fraction, *N* number, *MUGA* multi-gated acquisition, *MRI* magnetic resonance imaging

^a^Calculated from chi-square tests assessing differences in outcomes between LVEF categories. Statistical tests were two-tailed

**Fig. 2:**
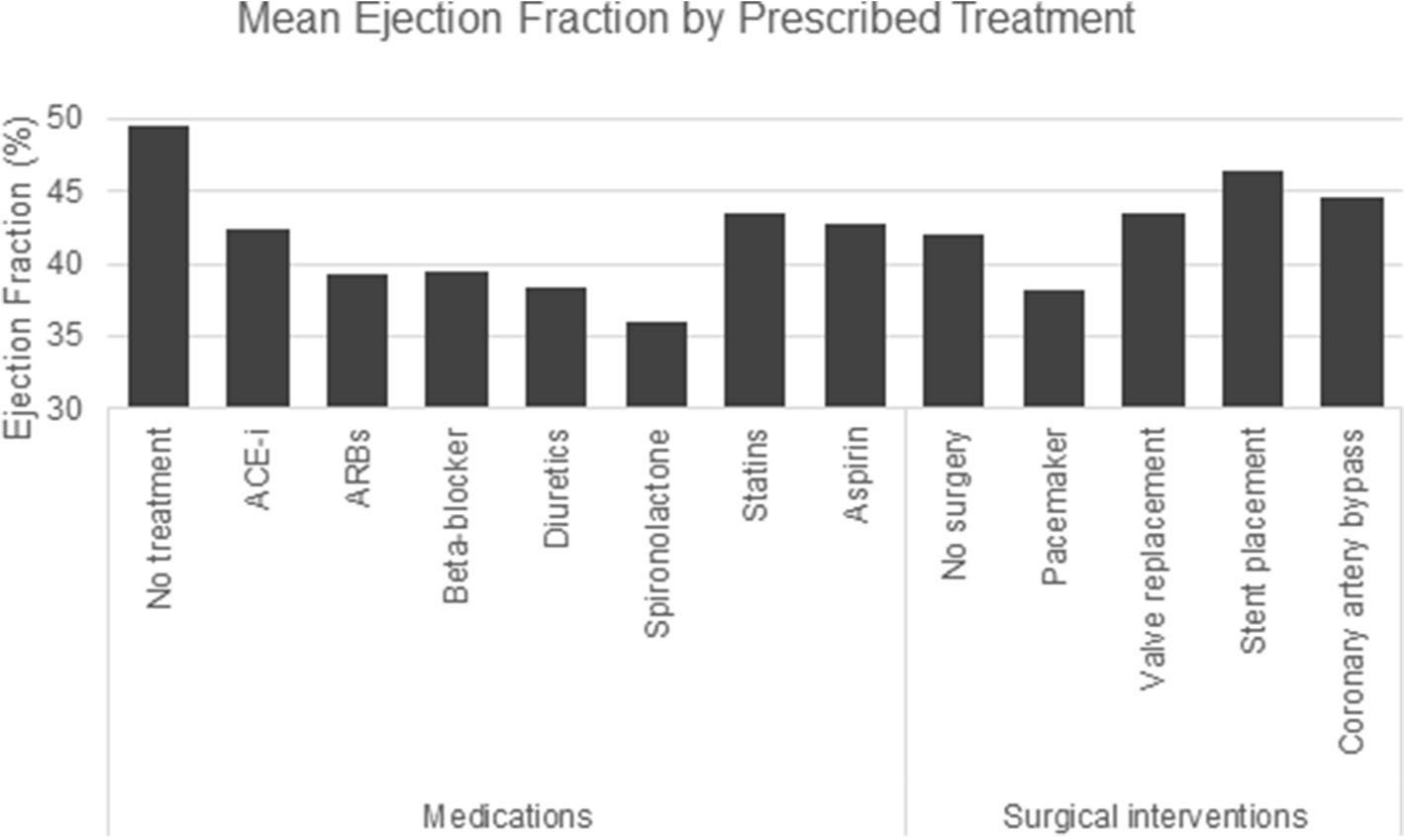
Mean ejection fraction by prescribed treatment among 80 childhood and young adult cancer survivors with low left ventricular ejection fraction

Fifty-one survivors (63.8%) were prescribed at least one cardiovascular medication. The most common medications represented included 37 (46.3%) who were prescribed ACEI/ARB, 30 (37.5%) who were prescribed beta-blockers, and 28 (35.0%) who were prescribed statins. Twelve (15.0%) survivors received at least one surgical intervention – with 11 (13.8%) receiving a pacemaker or ICD and 7 (8.8%) undergoing a valve replacement (Table 4). Receiving any medication (*P* < 0.001), specifically ACEI/ARB (*P* = 0.010), beta-blockers (*P* < 0.001), diuretics (*P* = 0.001), aldosterone antagonists (*P* = 0.015), or aspirin (*P* = 0.036) was significantly varied by group, as was receiving a pacemaker or ICD (*P* = 0.031). Mean LVEFs by prescribed cardiac medications ranged from 35.9% for aldosterone antagonists to 43.5% for statins. Mean LVEFs by surgical interventions ranged from 38.1% for pacemaker or ICDs to 46.5% for stent placement (Fig. 2).

**Table 4:** Cardiac treatments among 80 childhood and young adult cancer survivors with low left ventricular ejection fraction

Treatments	N (%)	LVEF 50–54.9% *N* = 38 (47.5%)	LVEF 41–49.9% *N* = 18 (25.0%)	LVEF < 41% *N* = 24 (30.0%)	*P*-value^a^
Medications					
Any medication	51 (63.8)	16 (42.1)	13 (72.2)	22 (91.7)	< 0.001
ACEI/ARB	37 (46.3)	12 (31.2)	8 (44.4)	17 (70.8)	0.010
Beta-blocker	30 (37.5)	7 (18.4)	6 (33.3)	17 (70.8)	< 0.001
Diuretics	19 (23.8)	5 (13.2)	2 (11.1)	12 (50.0)	0.001
Aldosterone antagonists	7 (8.8)	1 (2.6)	2 (11.1)	4 (16.7)	0.015
Statins	28 (35.0)	9 (23.7)	8 (44.4)	11 (45.8)	0.130
Aspirin	22 (27.5)	6 (15.8)	5 (27.8)	11 (45.8)	0.036
Surgical interventions					
Any intervention	12 (15.0)	4 (10.5)	2 (11.1)	6 (24.0)	0.260
Pacemaker/ICD	11 (13.8)	3 (7.9)	1 (5.6)	7 (29.2)	0.031
Valve replacement	7 (8.8)	3 (7.9)	2 (11.1)	2 (8.3)	0.921
Stent placement	4 (5.0)	2 (5.3)	1 (5.6)	1 (4.2)	0.974
Coronary artery bypass	4 (5.0)	2 (5.3)	0 (0.0)	2 (8.3)	0.469

*LVEF* Left ventricular ejection fraction, *N* Number, *ACEI* Angiotensin converting enzyme inhibitor, *ARB* Angiotensin receptor blocker, *ICD* Implantable cardioverter-defibrillator

^a^Calculated from chi-square tests assessing differences in outcomes between LVEF categories. Statistical tests were two-tailed

### <41% LVEF group

Survivors that fell into the < 41% LVEF group (*N* = 24) had a median lowest LVEF of 32.5% (IQR: 28.0–37.5) and a median time from diagnosis of 33.0 years (IQR: 23.8–42.4). This reflected a relatively longer follow-up time, a measure that was significantly varied by group (*P* = 0.002). These survivors were sicker overall, and were more likely to have left ventricular hypertrophy, left atrial enlargement, valvular disease, coronary artery disease, and congestive heart failure than other groups. Many of these survivors were symptomatic and were referred to and managed by a cardiologist, where follow-up intervals were as narrow as a few months. Some of these survivors were noted to see multiple cardiologists at outside institutions, often closer to their place of residence. Some received Holter monitors depending on the severity and characteristic of symptoms. Many survivors reported fatigue, chest pain, shortness of breath, and palpitations. Several survivors with advanced disease were hospitalized for cardiac symptoms. Diabetes mellitus was more prevalent in this group as well, and two died of cardiac complications during the follow-up period.

Some of the survivors in the < 41% LVEF group presented to acute care with cardiac symptoms, at times requiring admission to the cardiac intensive care unit and administration of inotropic agents. While the majority progressed gradually, the decision to treat these survivors was usually less ambiguous than it was for the two other low-LVEF groups. Treatments generally began with ACEI/ARB or beta-blockers. These survivors were more carefully consulted about the importance of exercise, diet, and electrolyte control. At least one patient struggled to keep up with care due to socioeconomic factors. All female survivors of reproductive age were advised to notify the medical team if they became pregnant. Another patient put off oral contraceptives as it complicated her blood pressure control. Further, cardiology input was solicited for survivors who developed subsequent cancers with regards to therapeutic options.

### 41–49.9% LVEF group

Survivors that fell into the 41–49.9% LVEF group (*N* = 18) had a median lowest LVEF of 46.5% (IQR: 44.3–48.0) and a median time from diagnosis of 26.3 years (IQR: 24.9–30.5). There was more heterogeneity in the presentation of disease among these survivors than among those in the lowest LVEF category. This variability may have contributed to a slightly higher utilization of additional cardiac testing, such as echocardiogram stress tests, pharmacological nuclear stress tests, MUGA scans, and angiograms.

In the setting of symptoms, treatments generally began once LVEF dropped below 50%, and was often titrated to maximum tolerable therapeutic doses. At times, however, treatment was put off if the patient was asymptomatic and further testing did not raise any concerns. Some survivors that initially presented to cardiology with symptoms and low-LVEF were later determined to be experiencing an acute episode, with a resolution of symptoms and improved LVEF thereafter. One patient in this group was diagnosed with low-LVEF at 22 weeks of pregnancy. This patient was given a Holter monitor, tested monthly with echocardiograms and for brain natriuretic peptide, given beta-blockers and diuretics as needed, and was referred to a high-risk obstetric medical group. Another patient was referred to cardiology specifically for determining whether amphetamines for attention deficient disorder was appropriate given his borderline low-LVEF. Further, several younger survivors described keeping up with their appointments and medications as stressful and getting in the way of living a normal life.

### 50–54.9% LVEF group

Survivors that fell into this category in our study had a median lowest LVEF of 52.5% (50.6–53.1) and a median time from diagnosis of 23.7 (15.5–31.0) years. These survivors were the least likely to be symptomatic (21.1%), most likely to have echocardiogram follow-up intervals of > 1 year (28.9%), and were the least likely to receive medications (42.1%) or surgical interventions (10.5%).

Preventative treatments such as ACEI/ARB or beta-blockers were implemented in 42.1% of these survivors. Because many of these survivors were young and did not have hypertension at baseline, doses were slowly titrated and discontinued if debilitating hypotensive symptoms presented. Like all groups, survivors were consulted on diet and exercise. Survivors not requiring immediate treatment were informed of their cardiac risk due to their cancer treatment and of the importance of long-term monitoring to detect potential issues.

## Discussion

To our knowledge, this is the first study to describe management approaches for monitoring and treating low-LVEF among adult survivors of CAYA cancer with a history of anthracycline chemotherapy or thoracic RT. Qualitatively, we found that management approaches largely followed guidelines for low-LVEF in the general population. Several preventative and screening guidelines for this population are available in the literature, with a distinct lack of high-level evidence for treatments [11, 18]. As a result, clinicians often rely on guidelines from the American Heart Association and American College of Cardiology (AHA/ACC) or ESC, which are designed for the general population [12, 19]. However, considering the relative young age and high prevalence of both comorbidities and subsequent neoplasms in this population, general guidelines may not be adequate.

One notable difference we observed was the relatively low use of aldosterone antagonists including spironolactone (8.8%) – which was generally lower than other heart failure medications – despite compelling data supporting its use to decrease mortality [20]. It is possible that aldosterone antagonists’ hormonal side effects may have contributed to clinicians prescribing this class of medications less frequently than other heart failure medications in this young population. Spironolactone binds the androgen receptor and induces gynecomastia in men and amenorrhea in premenopausal women [20]. Newer aldosterone antagonists with less hormonal side effects have been introduced [20], however, some clinicians may be conditioned to avoid this class all-together in younger patients.

We observed ambiguity in the management of survivors with higher LVEFs. The 41–49.9% (borderline) LVEF group had highest rate of obtaining echocardiogram and pharmacological stress tests and MUGA scans, while the 50–54.9% LVEF group had the highest rate of receiving at least one test overall, ECGs, and cardiac MRIs – the latter of which provides similar information as echocardiograms (valve pathology, ejection fractions) but with better image quality, more accuracy, and higher cost [21]. This suggests that clinicians were searching for more evidence to inform their treatment decisions. The uncertainty among clinicians for managing these patients mirrors what has been reported for survivors with borderline LVEF in the general population, where firm evidence-based management guidelines are lacking [22]. That said, there is evidence that aldosterone antagonists [23, 24] and ARB [25] can reduce heart failure hospitalizations among those with borderline LVEF; future studies of their use in the survivorship setting are needed.

We also observed a subgroup of survivors who had low-LVEF as an acute episode around the time of treatment, with improved LVEF on subsequent echocardiograms. For asymptomatic survivors, medical records reflected uncertainty in management. Spontaneous improvement in LVEF has been reported in the general population, with the recommendation that patients be kept on the same medical regimen [26, 27]. However, recommendations for cancer survivors who may have had an acute low-LVEF episode – which is believed to manifest through a mechanism that is distinct from chronic cardiomyopathy – are sparse [28].

Lastly, we observed survivors who were under consideration for additional anthracycline and/or thoracic RT for a secondary cancer. For survivors with low-LVEF, clinicians in this study weighed the benefit that additional cancer therapy would provide with the risk that it may accelerate existing heart problems. For the general population, routine measurement of LVEF before the administration of anthracyclines is generally believed to have low utility, as low-LVEF is a relatively rare condition in otherwise healthy patients [29–31]. However, considering that childhood and young adult cancer survivors have a much higher risk of cardiac complications [8] with guidelines that recommend regular cardiac screenings [9], left ventricular function is extremely relevant. Still, with lack of specific recommendations, management of these survivors varies. Additional guidelines that consider a patient’s heart health and cancer burden when deciding to administer subsequent anthracyclines or thoracic RT would be useful.

Our analysis is limited as it only describes management practices at a single institution. Future research of management practices at other institutions could elucidate other problems unique to this population, ultimately providing the framework for future guidelines. Until these guidelines become available, cancer survivors with cardiomyopathy secondary to treatment should continue to be managed according to guidelines for the general population. Further, we have also found that enrollment in a specialized survivor clinic improves patient adherence to guideline-concordant care, a finding that has been shown by other institutions for screening [32]. Clinicians with experience dealing with issues unique to cancer survivors can help fill any gaps that current management guidelines may have.

## Conclusion

In summary, our results suggest that the management of childhood and young adult cancer survivors with low-LVEF largely follows guidelines designed for the general population. However, uncertainty regarding treatment for survivors with borderline low-LVEF, those with acute episodes of low-LVEF, or those undergoing additional treatment for a subsequent malignancy continues to be a challenge for clinicians treating this high-risk population. Future research should consider a focus on these scenarios. We hope this single institution experience on common practice approaches can be used to inform future clinical trials and formal guidelines concerning best practices for cancer survivors with heart failure secondary to cancer treatment.

## Data Availability

Statistical code available upon request.
