# Correction: Approaches for monitoring and treating cardiomyopathy among cancer survivors following anthracycline or thoracic radiation treatment

**DOI:** 10.1186/s40959-022-00141-2

**Published:** 2022-08-19

**Authors:** Arash Delavar, Catherine Boutros, Dana Barnea, Wendy L. Schaffer, Emily S. Tonorezos

**Affiliations:** 1 Department of Medicine, Memorial Sloan Kettering Cancer Center, New York, NY USA; 2 University of California San Diego School of Medicine, La Jolla, San Diego, CA USA; 3 City University of New York School of Medicine, New York, NY USA; 4 Tel Aviv Sourasky Medical Center, Tel Aviv, Israel; 5 Division of Cancer Control and Population Sciences, National Cancer Institute, National Institutes of Health, Rockville, MD USA; 6 Ofce of Cancer Survivorship, National Cancer Institute, 9409 Medical Center Drive, Rockville, MD 20850 USA

**Correction: Cardio-Oncology 8, 11 (2022)**

**https://doi.org/10.1186/s40959-022-00138-x**

Following publication of the original article [1], the authors identified an error in the author name of Wendy L. Schaffer.

The incorrect author name is: Wendy L. Schaffer MD

The correct author name is: Wendy L. Schaffer

The author group has been updated above and the original article [1] has been corrected.
